# Structural transitions in full-length human prion protein detected by xenon as probe and spin labeling of the N-terminal domain

**DOI:** 10.1038/srep28419

**Published:** 2016-06-24

**Authors:** Sunilkumar Puthenpurackal Narayanan, Divya Gopalakrishnan Nair, Daniel Schaal, Marisa Barbosa de Aguiar, Sabine Wenzel, Werner Kremer, Stephan Schwarzinger, Hans Robert Kalbitzer

**Affiliations:** 1 Institute of Biophysics and Physical Biochemistry and Centre of Magnetic Resonance in Chemistry and Biomedicine (CMRCB), University of Regensburg, 93040 Regensburg, Germany; 2 Research Center for Bio-Macromolecules and Department of Biopolymers, NW1/BGI, University of Bayreuth, 95447 Bayreuth, Germany

## Abstract

Fatal neurodegenerative disorders termed transmissible spongiform encephalopathies (TSEs) are associated with the accumulation of fibrils of misfolded prion protein PrP. The noble gas xenon accommodates into four transiently enlarged hydrophobic cavities located in the well-folded core of human PrP(23–230) as detected by [^1^H, ^15^N]-HSQC spectroscopy. In thermal equilibrium a fifth xenon binding site is formed transiently by amino acids A120 to L125 of the presumably disordered N-terminal domain and by amino acids K185 to T193 of the well-folded domain. Xenon bound PrP was modelled by restraint molecular dynamics. The individual microscopic and macroscopic dissociation constants could be derived by fitting the data to a model including a dynamic opening and closing of the cavities. As observed earlier by high pressure NMR spectroscopy xenon binding influences also other amino acids all over the N-terminal domain including residues of the AGAAAAGA motif indicating a structural coupling between the N-terminal domain and the core domain. This is in agreement with spin labelling experiments at positions 93 or 107 that show a transient interaction between the N-terminus and the start of helix 2 and the end of helix 3 of the core domain similar to that observed earlier by Zn^2+^-binding to the octarepeat motif.

Prion diseases or transmissible spongiform encephalopathies (TSEs) are a family of rare progressive neurodegenerative disorders that affect both humans and animals alike. Examples of prion-causing diseases are bovine spongiform encephalopathy (BSE) in cattle and Creutzfeldt-Jakob disease (CJD) in humans^1^,^2^,^3^,^4^,^5^. These conditions are associated with the accumulation of an oligomeric conformational scrapie isomer PrP^Scr^ of the host-encoded monomeric prion protein PrP^c^. According to the “protein-only” hypothesis^6^, it is suggested that the conformational isomer of PrP^Scr^ is able to convert other isoforms to the infectious isomer in an autocatalytic process. A detailed knowledge of this conformational transition is mandatory for explaining the molecular basis for prion diseases^7^.

Mammalian PrP^c^ consists of two domains, a flexibly disordered N-terminal segment and a globular C-terminal domain containing three-helices and two short antiparallel-strands^8^. In addition, it contains a single disulfide bond linking cysteine residues at positions 179 and 214 between α2 and α3, which stabilize the folded structure of the normal protein. It has been suggested that the conformational state of the prion protein disulfide bond may have implications for correct maturation and function of this protein^9^,^10^. Prionic diseases were identified in many species of mammals, that also show a transmission specificity and infectivity of prions inter species^11^. Species barriers seem to occur in the majority of cases, but there are indications that species barrier crossing to infect another species requires specific changes of the amino acid sequence. Many groups have been focusing their research in discovery of these critical regions of the primary sequence of the protein, using modern techniques^12^,^13^,^14^,^15^,^16^,^17^,^18^. A region especially important for species differences in infectivity is the loop between β-strand 2 and α-helix 2 from residue 166 to 172 that occurs in two conformational states, in one state it contains a 3_10_-helix, in the other state a type-I β-turn. The population of the two states in different species mainly depends on the existence of a tyrosine or phenylalanine residue in position 169^18^. For oligomerisation and fibril formation presence of the characteristic sequence motif AGAAAAGA (residues 113–120) is necessary^19^ which is part of the unfolded N-terminus of the prion protein and is located just in front of the well-folded core. After oligomer formation the residues of the AGAAAAGA motif are immobilized whereas all seven tryptophan residues located in the N-terminal range from amino acid 31 to 99 including the four tryptophan residues of the copper binding octarepeat remain freely mobile^20^. There are also neuroprotective mutations such as G127V, which leads to a complete protection for Kuru and CJD^21^.

Proteins are not rigid entities. For proper function they have to exist in several conformational states (“excited states”) with higher free energies than the ground state. This has been shown exemplarily for proto oncogene Ras involved in cellular signal transduction^22^. A pivotal role for the corresponding structural transitions play fluctuating low density zones and cavities in the protein^23^. These regions often can be identified by their typical strong, non-linear pressure response in high-pressure NMR spectroscopy^24^; the corresponding structural transition can be characterized by thermodynamic analysis of the data. Such a study has been reported for the human and the Syrian hamster prion protein^15^,^25^,^26^ where four different states of the folded core with differences of Gibbs free energies Δ*G*^0^ of approximately 3, 11, and 19 kJ/mol could be defined. The first transition would describe a transition *N*_1_ to *N*_2_ in the natively folded ensemble with a relative population of 0.3^26^.

An alternative method to detect structural fluctuations is based on the interaction of a protein with xenon detected by NMR-spectroscopy. Xenon is a noble gas that specifically recognizes hydrophobic cavities in macromolecules^27^,^28^,^29^,^30^. The interaction between xenon and the atoms of the protein are established uniquely through London dispersion forces. It can also bind to cavities that in the ground state are too small to host a xenon atom, but open up transiently. Thus, the corresponding rare conformational states can be detected by xenon binding. This has been shown earlier for the mutant HPr(I14A) of the histidine containing protein that contains a hydrophobic cavity that is filled by a side chain of a leucine residue in the ground state^31^. Here, xenon recognizes and stabilizes the lowly populated conformation of the wildtype protein that opens up to encompass a xenon atom^32^. Correspondingly, in the present study xenon is used to probe structural transitions coupled to fluctuations of hydrophobic cavities in *hu*PrP(23–230) and correlate them with the transitions detected by high pressure NMR-spectroscopy.

In a first approximation, typical NMR parameters such as chemical shifts, relaxation times, and missing NOEs indicate that the N-terminal segment is mobile and disordered. However, already in the early paper by Zahn *et al*.^8^ the authors realized from an analysis of their NMR data that between the unfolded part of the protein and the well-folded part at least transient interactions have to exist since in the folded part chemical shift changes were observed when the N-terminal residues were removed. These chemical shift changes comprise residues 187 to 193 in the C-terminal part of helix 2 and the eight successive residues from position 219 to 226 in the C-terminal part of helix 3. These regions, defined by chemical shift differences between full length PrP(21–230) and truncated PrP(120–230), are part of a region from amino acids 186 to 226 with an exceptionally large pressure coefficients. These pressure coefficients change significantly when the N-terminal part is truncated, indicating (transient) interactions between the N-terminal domain and the core domain. Especially G127, E168, H187, T192, E207, E211, and Y226 show significant differences in the NMR detected pressure response after removal of the N-terminus^26^. There is a number of additional evidence that there is some functional and conformational coupling between the N-terminal domain and the core domain, for instance, elimination of a segment between the N- and C-terminal domains in transgenic mice results in an embryonic lethal phenotype^33^,^34^. A most interesting region in the N-terminal domain is the octarepeat region (PHGGGWGQ)_4_ from amino acid 60 to 91 that is able to bind Cu^2+^- and Zn^2+^-ions^35^,^36^,^37^. Binding of these ions induces PrP endocytosis^38^, inhibits *in vitro* fibril formation^39^ and suppresses PrP^Scr^ amplification^40^. Recently, double electron-electron resonance (DEER) experiments showed multiple equilibria of the N-terminus relative to the core domain of mouse PrP as detected by spin labels at position of Q61 and T200. Zn^2+^-binding to the octarepeat induces an almost complete shift of conformational equilibrium to a state where the octarepeat region is close to T200 at the start of helix 3 suggesting that this closed conformation protects the PrP from fibril formation^41^. Zn^2+^-binding to the octarepeat itself leads to chemical shift and line widths changes in the region encompassing F140 to Y145 in loop 1, Q171 to T182 at the start of helix 2, and K203 to Y217 in helix 3^41^.

For getting more experimental information about such an conformational equilibrium, we introduced spin labels just after the octarepeat in position 93 and in front of the AGAAAAGA motif in position 107 and observed their relaxation enhancement effects on the [^1^H, ^15^N]-HSQC spectra of human prion protein as presented in the following.

## Results

### Identification of xenon binding sites

When Xenon binds to hydrophobic cavities its binding can be detected by chemical shift perturbations and/or cross peak volume changes in [^1^H-^15^N] heteronuclear single quantum coherence (HSQC) spectra of a protein. [^1^H-^15^N]-HSQC spectra of ^15^N-enriched huPrP(23–231) were used to localize xenon binding sites on the protein structure at different xenon concentrations defined by slight xenon overpressure^28^. A reference [^1^H-^15^N]-HSQC spectrum was measured and assigned based on the data deposited in the biological magnetic resonance data bank (BMRB#4402) as well as in-house assignments^26^. Upon subjecting the samples to xenon pressures of 0.2, 0.4, 0.8 and 1.4 MPa (see Materials and Methods), corresponding to xenon concentrations of 8.8, 17.6, 35.2, and 61.6 mM in the protein solution respectively^28^, for a number of amide resonances changes of their chemical shift positions as well as their signal intensities/volumes were observed (Table 1). This demonstrates that the xenon atoms dissolved are interacting with the prion protein in a site-specific, ligand-like fashion in solution.

**Figure 1:**
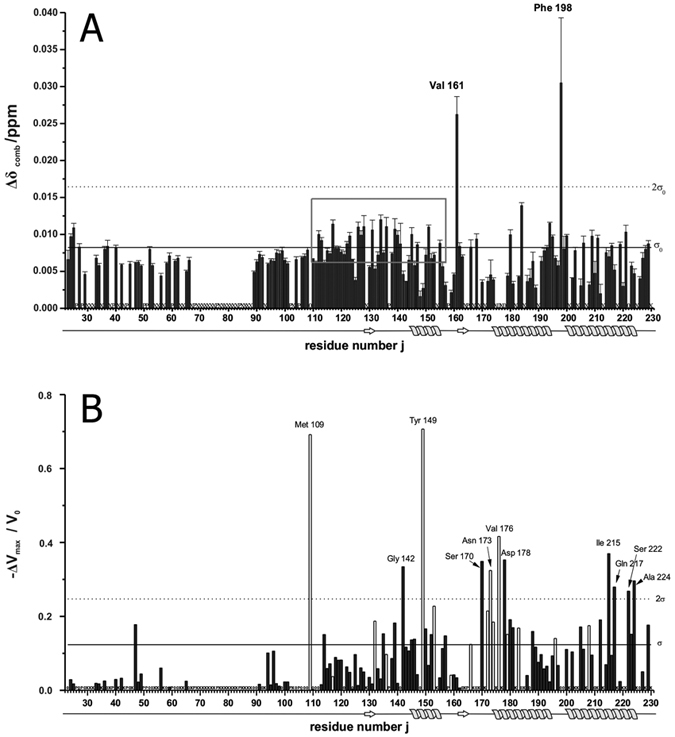
Chemical shift and cross peak volume perturbation by xenon binding. Maximum combined chemical shift changes Δ*δ*_comb_ (**A**) and relative changes of the cross peak volumes −Δ*V*_max_/*V*_0_ = (*V*_0_ − *V*(*c*_max_))/*V*_0_ (**B**) observed in the [^1^H, ^15^N]-HSQC spectra of ^15^N enriched *hu*PrP(23–230) are plotted as a function the residue number *j* at 293 K. *V*_0_ is the cross peak volume in the absence of xenon and *V*(*c*_max_) the volume at the maximum xenon concentration *c*_max_ of 61.6 mM. Solid line, standard deviation *σ*_0_ to zero, dotted line, 2*σ*_0_. P marks prolines, X other residues that are not visible or are not assigned in the spectra, 0 residues where satisfactory values could not be obtained. The error bars correspond to the standard errors. White bars represent residues that do not show saturation behaviour at the highest xenon concentration.

Figure 1A summarizes the combined chemical shift differences Δ*δ*_comb_ of each observable residue j as a function of the protein primary structure. The secondary structure of the prion protein is indicated at the bottom of the figure. Many residues show a significant chemical shift response by xenon binding above the standard deviation *σ*_0_ indicative for a generalised conformational change of the protein upon xenon binding (Table 1). This also includes residues such as K24 and R25 located at the start of the unstructured N-terminus and residues in or close to the AGAAAAGA (113–120) motif required for fibril formation. In the folded core structure of the prion protein xenon effects are observed in all secondary structure elements of PrP (Table 1). The region showing an almost continuous combined chemical shift difference above *σ*_0_ is highlighted in Fig. 1 by a grey rectangle. This region starts with M112 and ends with H155 including the amino acids of the preceding flexible N-terminus as well as the first β-strand and α-helix of the folded core. Chemical shift responses above 2*σ*_0_ are observed for V161 and F198 upon xenon binding (Fig. 1).

In addition to the chemical shift perturbations observed upon xenon binding a reduction of the signal volumes of the cross-peaks in the ^1^H-^15^N HSQC spectra was detected. Figure 1B displays the maximum relative volume changes induced by the highest xenon pressure used in this study as a function of the position in the amino acid sequence. The solid line again represents the standard deviation *σ*_0_, the dotted line 2*σ*_0_. Residues with a volume change larger than *σ*_0_ are summarized in Table 1. Values above *σ*_0_ can be considered as significant, values above 2*σ*_0_ as highly significant. Volume changes above 2*σ* are seen for M109 located in the unfolded N-terminal region as well as for residues G142 located in loop 1 and Y149 in α-helix 1 of the well-folded core. There are two clusters of strongly affected amino acids, one at the start of helix 2 (S170, N173, V176, D178), one at the end of helix 3 (I215, Q217, S222, A224) (Fig. 1). In addition, a few new signals in the [^1^H-^15^N]-HSQC spectra appear that in the absence of xenon have not been observed and are therefore not assigned.

**Figure 2:**
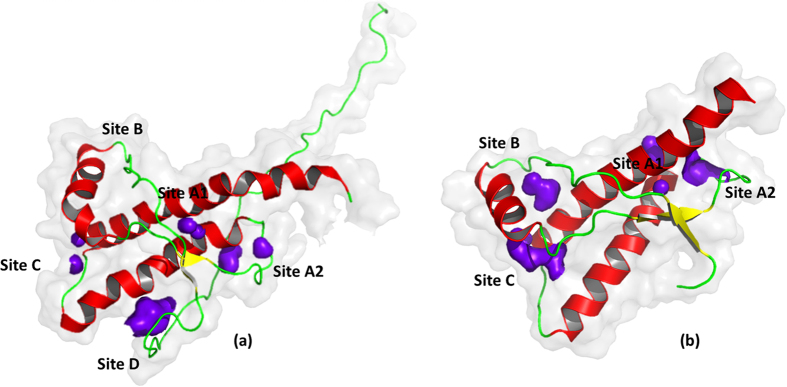
Cavities and pockets in human prion protein structures. Cavities were calculated with a probe radius of 0.12 nm in the huPrP structure deposited as (**a**) 2KUN (20^th^ structure of the bundle) and (**b**) 1QM2 in the protein data base, respectively. The surface of the cavities is represented in violet. For more details see Table 2.

NMR structures published of the human prion protein exhibit a number of hydrophobic cavities in the well-folded core (amino acids 125 to 231) but the full length prion protein may also transiently form new cavities when the presumably disordered N-terminus gets in contact with the compactly folded part of the protein. The cavities were analysed with the program CASTp in two sets of 20 NMR structures each published by Zahn *et al*.^8^ (pdb accession code 1QM1) and Ilc *et al*.^42^ (pdb accession code 2KUN), respectively. The size and shape of the cavities calculated by the program depends on the size of the probe that should fit into the cavity. We used two different probes, one with the size of a water molecule (radius 0.12 nm) and one with the size of a xenon atom (radius 0.22 nm). While the first NMR data set only contains coordinates of the folded core part of the structure (amino acid 125 to 228), the second data set provides additional information about the N-terminal amino acids (amino acid 90 to 227). It also has a point mutation at position 212 (Q to P), a mutation that is not expected to change the number or the properties of the cavities compared to the wild type. Using a water molecule as probe, it was found that cavities are not conserved among and within structural bundles as evidenced by cavity B shown in Fig. 2. An exception is cavity C that is present in all cases. Using a xenon atom as a probe the number of cavities that can accept a xenon atom without steric clashes is significantly reduced in the structural bundles. Cavity B is not large enough to host a xenon atom in any of the structures of the two data sets. Nevertheless, our experimental data support its (transient) existence in solution, since strong local effects induced by xenon binding are observable. A fifth cavity D is formed by the N-terminal part of the protein in some structures deposited in the data set 2KUN. This part of the structure is not included in data set 1QM1 and can therefore not be analyzed (Fig. 2). However, as a rule, the total volumes of most of the cavities found to fit a water molecule are larger than the corresponding volume of a sphere with the radius of a xenon atom with a volume of 0.045 nm^3^ (Table 2), indicating that these cavities could adopt xenon after a suitable change of their shapes.

In first approximation the largest chemical shift or cross peak volume changes are to be expected close to xenon binding sites where xenon binding induces local conformational changes. These residues listed in Table 2 correlate well with the residues forming the surface of the cavities detectable in the two bundles of NMR structures (1QM1 and 2KUN). A good correlation is observed but not all residues delineating a cavity experience chemical shift changes above *σ*_0_. This is to be expected since not all residues will be in direct contact with the xenon atom and/or involved in the required shape change of the cavity under consideration. More than 50% of the 65 residues showing significant effects >*σ*_0_ after xenon binding are part of a cavity surface (Tables 1 and 2), most of the other residues contribute to the second order shell of a cavity. From the twelve residues with xenon effects >2*σ*_0_ nine are located close to a cavity, eight of them directly contribute atoms to the surface of the cavities.

### Affinity of individual cavities for xenon

**Figure 3:**
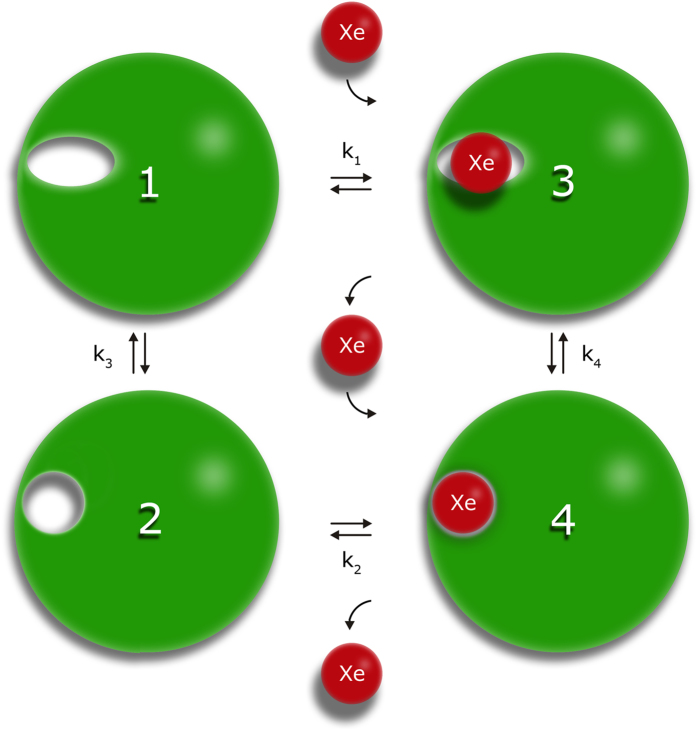
Xenon binding in a two state model. In state 1 the cavity is too small to accommodate a xenon atom, in state 2 it has opened up. The equilibrium constants *k*_i_ are defined as *k*_1_ = [3]/([Xe][1]), *k*_2_ = [4]/([Xe][2]), *k*_3_ = [1]/[2], *k*_4_ = [3]/[4].

Since we experimentally demonstrated xenon binding to all cavities, either (1) the NMR structures do not represent the cavities with sufficient reliability and all cavities are always large enough to accept a xenon atom, or (2) the cavities exist in a second transient state of the protein, where a xenon atom can be incorporated. A special case is cavity D that is only formed when the N-terminus is in contact with the main folded structure. This interaction has to be transient, since it is only found in a small number of the structures. A satisfactory fit of the experimental data was only possible assuming an equilibrium between an open and a closed state of the cavities. However, the quality of the data does not allow a stable fit including the equilibrium constant for the open and closed state as additional free parameter. In a first approximation for this equilibrium constant the statistical distribution of the states, where the cavities are large enough to accommodate a xenon atom in the solution NMR structural bundles obtained by restraint molecular dynamics, can be used as an estimate of the occupancy of the states where a xenon atom can be incorporated (open state 1) and where not (closed state 2). The minimum scheme for such a dynamical equilibrium is depicted in Fig. 3. Cavity D is only formed by the interaction of the core structure with the N-terminus (corresponding in the model of Fig. 3 to the open state) and disappears when the contact is lost (“closed” state). For reasons of simplicity the model assumes that xenon binding to different cavities is independent. When xenon cannot bind to the closed state since it does not fit into the corresponding cavity or the xenon binding state does not exist in one state, the xenon binding constant *k*_1_ can be set to zero.

**Figure 4:**
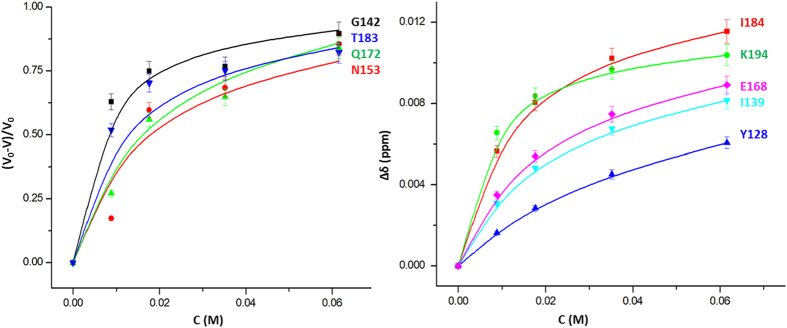
Dependence of chemical shift and cross peak volumes on the xenon concentration. The sample contained 0.25 mM of ^15^N-enriched huPrP(23–230) in 5 mM acetate buffer, 0.1 mM DSS, pH 4.5 in 90% ^1^H_2_O/10% ^2^H_2_O. Temperature 293 K. For residues typical for the various cavities (left) the relative intensity changes −Δ*V*/*V*_0_ and (right) the combined chemical shift changes Δδ_comb_ are plotted as a function of the free xenon concentration *c*. The curves shown were calculated with the parameters of Table 3, only the chemical shifts in the different states are free fit parameters.

The response of the chemical shifts and the cross peak volume changes on xenon binding depends on the time scale of the transitions between the four different states. It is assumed that in the closed state xenon cannot bind to the cavities. When the exchange is fast on the NMR time scale for all four possible transitions depicted in Fig. 3 (1/*τ*_ij_ ≫ |Δ*ω*_ij_|, with *τ*_ij_ being the exchange correlation times for the exchange between states i and j and Δ*ω*_ij_ corresponding to the associated chemical shift differences), the dependence of the observed chemical shift changes on the xenon concentration can be fitted with eq. 16. They are dependent on the association constant *k*_2_^i^ of xenon to the open state of a given binding site i and the equilibrium constant *k*_3_^i^ for the equilibrium between open and closed state. From the largest chemical shift change, a lower limit for the exchange correlation time can be estimated as 35 ms for the equilibrium between the open and closed state. Figure 4 shows examples for chemical shift changes of residues typical for the different cavities. The curves were fitted with the association constants given in Table 3 that describe the thermodynamic equilibrium for all residues assignable to a given cavity (Table 2). Only the chemical shifts in the different states had to be fitted individually since they depend on the environment of an individual spin in the absence and presence of xenon, respectively.

For some residues no chemical shift changes but only a decrease of their cross peak volumes *V*(c) is observed with increasing xenon concentrations c. Such behaviour could be due to an increased *T*_2_-relaxation causing a reduction of the INEPT-transfer and/or by a two-site slow exchange process. Since overall only small changes of the cross peak line widths were observed in the HSQC spectra, the comparatively large volume reductions can only be explained by the second process. When the exchange correlation time is slow in the NMR time scale for a given residue between the open and closed state and fast for the xenon interaction itself, eq. 9 can be applied. A common fit of the relative volume changes of residues close to a given cavity gives association constants that within the limits of error are identical to those obtained from the fit of the chemical shift changes.

The obtained microscopic association constants *k*_2_ for xenon binding to the open state vary between 0.1 and 4.5 mM^−1^ (Table 3). However, the exact values depend on the probability of a cavity to be open and closed described by *k*_3_. As stated above the experimental data is not sufficient to simultaneously determine the equilibrium constant *k*_3_ with sufficient precision, but the fit of the data is consistent with the assumption, that the NMR structural bundles represent the occupancies sufficiently well. The apparent xenon affinity can be approximated by *k*_2_/(1 + *k*_3_) and is dependent on the opening probability of a xenon binding site. Small opening probabilities will decrease the apparent xenon affinities accordingly. The apparent xenon affinities calculated from the data given in Table 3 are of the order of 0.1 mM^−1^ corresponding to apparent dissociation constants of 10 mM and vary from cavity to cavity. However, since they are obtained from a fit of many different residues enclosing a given cavity their fit error is rather small and indicates that their differences are significant.

### Structure of the prion protein with xenon bound

**Figure 5:**
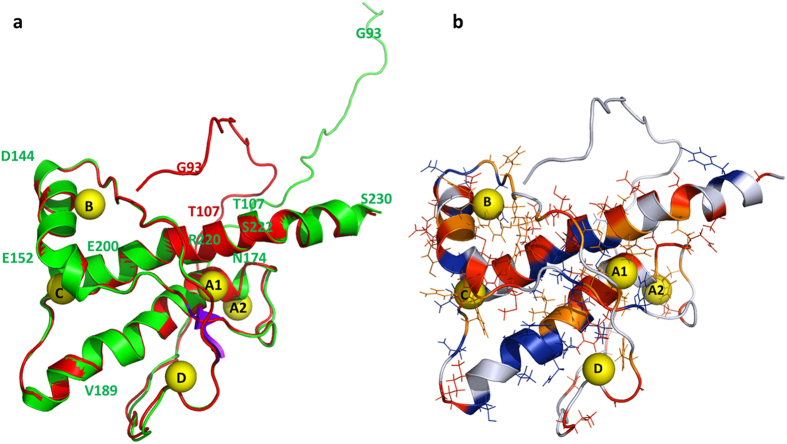
NMR-Structure of *hu*PrP with bound xenon. The NMR structure of *hu*PrP with xenon bound was calculated from the NMR data starting with the 20^th^ structure of 2KUN. (**a**) Overlay of the structure in the presence (red) and absence of xenon (green). (**b**) Lowest energy structure with residues showing significant effects induced by binding of xenon and/or by applying high pressure^15^,^22^. Residues showing only xenon effects > *σ*_0_ (red) and in addition high pressure effects > 2*σ*_0_ (orange). Residues showing high pressure effects > *σ*_0_ only are depicted in blue, residues not detectable (e.g. prolines) or with smaller effects are depicted in grey.

The structural model of the prion protein with xenon bound at the four cavities was calculated by placing xenon atoms in the four cavities, optimizing the structure by a simulated annealing approach on the basis of pseudo restraints obtained from the chemical shift and cross peak volume changes after binding of xenon. The obtained structures were refined in explicit water (for details see Materials and Methods). The lowest energy structure is shown in Fig. 5 together with the residues showing significant effects after binding of xenon. The structure with five xenon atoms bound is compared with the initial structure where some of the cavities are too small to accept a xenon atom. Xenon binding induces some structural rearrangements in the xenon binding cavities but only small global changes are required for accommodating the xenon atoms. The xenon atoms mainly interact with hydrophobic amino acid side chains and aromatic rings, in cavity A1 with V161 and F175, in cavity B with F141 and Y150, in cavity C with Y157 and F198, and in cavity D with L125, Y128, and Y162. Only cavity A2 does not show an interaction with such typical hydrophobic residues. Here the methylene groups of E168, S170, and N174 constitute the main interaction partners (see supplementary Table S1). Note that the positions of the first N-terminal residues in this figure and especially the location of G93 is quite arbitrary, since it is the result of the molecular dynamics refinement but is not supported by sufficient experimental restraints.

Figure 5 depicts those residues where significant pressure effects were reported in the absence of xenon^15^,^26^. As to be expected (see Introduction), they also cluster around the cavities and largely overlap with the residues that are sensitive to xenon binding.

### Detection of transient structural transitions by paramagnetic enhancement

**Figure 6:**
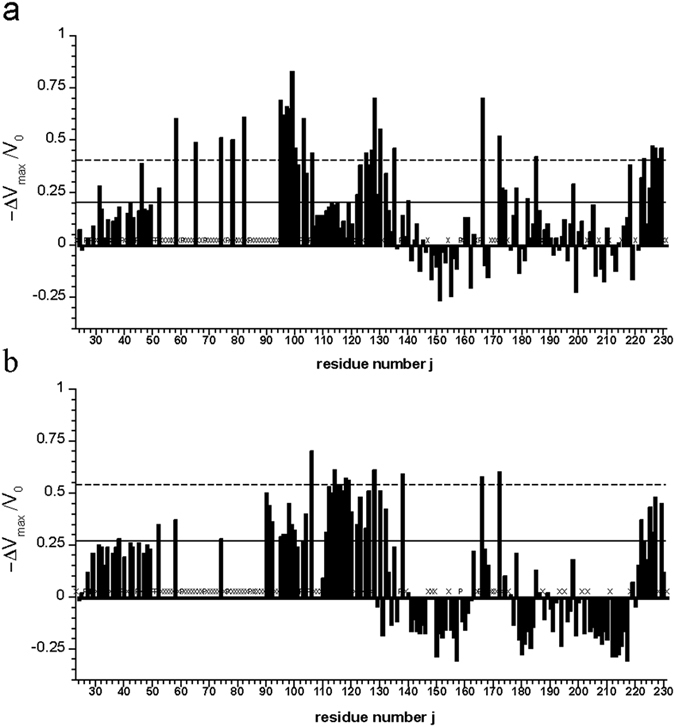
Paramagnetic effects in spin labelled full-length prion protein. The relative change −Δ*V*_max_/*V*_0_ = (*V*_0_ − *V*_max_)/*V*_0_ of cross peak volumes as a function of the sequence position j in [^1^H, ^15^N]-HSQC spectra of ^15^N-enriched huPrP(23–231) variants G93C (**a**) and T107C (**b**) labeled with methanesulfonothioate and maleimide based nitroxyl spin label, respectively. Solid and broken lines represent standard deviations *σ*_0_ and 2*σ*_0_. Proline residues are marked with P. Additionally invisible and unassignable residues in the spectra are marked with X. *V*_0_ corresponds to the cross peak volume after reduction of the spin label and *V*_max_ the cross peak volume after labeling (see Materials and Methods).

For obtaining information about transient interactions of the N-terminus with the globular domain, which also lead to formation of the transient cavity D, full-length prion protein variants with amino acid substitutions G93C and T107C were labelled with compounds containing unpaired electrons. In particular, the two different spin labels (1-oxyl-2,2,5,5-tetramethyl-∆3-pyrroline-3-methyl methanethiosulfonate and 1-oxyl-3-(maleimidomethyl)-2,2,5,5-tetramethyl-1-pyrrolidine) were applied in this study. Residues spatially close to the active spin label should show a reduction of their cross peak volumes due to enhanced *T*_2_- relaxation caused by strong electron-nucleus dipole-dipole coupling when compared to the reduced diamagnetic sample. Since the labeling reaction itself was not quantitative and since for the N-terminal residues no unique structure is to be expected, the analysis of the paramagnetic enhancement (PRE) was only done in a qualitatively manner by comparing differences in cross peak volumes of the paramagnetic and diamagnetic sample (after reduction of the spin label). The relative cross peak volume changes −Δ*V*/*V*_0_ = (*V*_0_ *−* *V*)/*V*_0_ were plotted as a function of the sequence position with the cross peak volumes *V* and *V*_0_ of active and reduced spin label, respectively. Solid and broken lines represent standard deviation *σ*_0_ and 2*σ*_0_ to zero of −Δ*V*/*V*_0_ the *V*/*V*_0_ (Fig. 6). The residues with significant cross peak volume changes are summarized in Table S2. They are assumed to be spatially close to the spin label in the time average but the averaging is non-uniform because of the *r*^−6^-dependence of the PRE effect.

Besides direct effects in the neighborhood of the spin label attached, both labeled variants show similar relative effects in the sequence positions 30–50, 120–135, ~165 and C-terminal region around residue 220–231. A number of residues show an increase of the cross peak volume in the labeled sample relative to the volume after reduction of the spin labels indicating that residual unattached spin label in solution enhances selectively the longitudinal relaxation of water protons and thus resulting in increased signal to noise ratio in the paramagnetic sample^43^.

**Figure 7:**
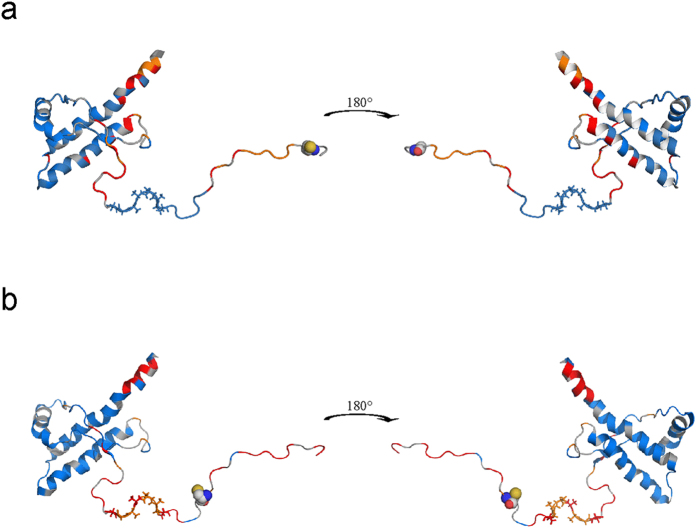
Mapping of paramagnetic effects on spin labeled full-length prion protein. Paramagnetic effects of MTSL and maleimide-linked nitroxyl spin label on huPrP variants G93C (**a**) and T107C (**b**), respectively. Very strong paramagnetic effects > 2 *σ*_0_ are colored in orange, strong effects > *σ*_0_ in red, small and negative effects < *σ*_0_ are colored in blue and unassigned residues in grey. The labeling position is displayed as spheres and the alanine-rich region in sticks. Mapping of the paramagnetic effects and *in silico* mutagenesis was done with Pymol (Schrödinger LLC) and the first structure of PDB entry 2KUN.

Most of the structures published by Ilc *et al*.^42^ deposited in PDB 2KUN ensemble cannot provide an explanation for the observed paramagnetic effects since the N-terminal domain is too distant from the globular domain. As an example we show the observed effects in the first structure of the bundle deposited in PDB 2KUN, where the residues that are significantly affected by the presence of the spin labels are given in a color code (Fig. 7). However, the structure of the prion protein selected for calculating the xenon containing structure (Figs 2 and 5) qualitatively explains many paramagnetic effects that cannot be explained by other structures of the flexible N-terminal domain of the same structural bundle, including e.g. the first structure of the same bundle, since the N-terminal domain apparently is too distant from the core domain (Fig. 7).

## Discussion

### Interactions of xenon in hydrophobic cavities

We were able to locate five binding sites for the xenon atoms by their NMR response to xenon binding. They correspond to cavities that are mostly large enough to accommodate water molecules and that are present in part of the NMR structures deposited in 2KUN and 1QM1, respectively. However, cavity D is only visible in some members of the structural bundle of 2KUN, since 1QM1 does not provide structural information about the corresponding flexible part of the N-terminal tail.

Site A_1_ shows a response of xenon binding at residues G131, S132, M134, V161, Y162, Y163, F175, V176, C179, C214, I215, Q127, Y218, E219, and R220 (Table 2). It is open for accepting a water molecule in almost all structures of the bundles deposited. Assuming that NMR data are sufficiently representative for the solution conformation of the proteins (what we will do in the following) a probability to be open *p*_open_ of 0.83 for water would follow resulting in a corresponding Δ*G*_H2O_ of 3.8 kJ mol^−1^ (Table 3). However, in most cases a xenon atom cannot enter the cavity reflected by an apparent xenon dissociation constant *K*_app_ of 12.5 mM and a Δ*G*_12_-value of −8.9 kJ mol^−1^. Site A2 is adjacent to this region and reacts on xenon binding by chemical shift and cross peak volume changes at R164, E168, S170, N171, N173, and N174. It can accept a water molecule in most of the structures (*p*_open_, 0.88; Δ*G*_H2O_ of 4.7 kJ mol^−1^). However, only in a few cases it is large enough to accept a xenon atom (*K*_app_ 14.6 mM; Δ*G*_12_ −4.2 kJ mol^−1^).

Of note, residues close to these two cavities are involved in prion diseases: E129M and D178N are characteristic mutations in *Fatal Familiar Insomnia* (FFI) while for *Creutzfeldt-Jakob* disease (CJD) mutations of the residues D178 and V180 are known to be involved in the disease development. The xenon interaction site A_1_ covers the polypeptide segment from 165–175, the β2-α2 loop, which was identified by the Wüthrich group in a whole number of prion structures to occur in different conformations in different species^8^,^18^,^44^,^45^ and which seems to be of crucial importance for disease progression in a mouse model^46^.

Xenon binding site B comprises residues I138, I139, F141, E146, D147, Y150, Y157, M205, R208, and Y209. It is open for a water molecule in all structures of the structural data set 1QM1 but only for 8 of the 20 structures of the 2KUN ensemble. Here, the two structural bundles differ significantly. The average Δ*G*_H2O_ is 2.1 kJ mol^−1^. In none of the structures a xenon atom can fit into the cavity (*K*_app_ 9.1 mM; Δ*G*_12_ < −9.0 kJ mol^−1^). None of the residues forming cavity B is known to be involved in any prionosis. However, the residues preceeding helix 1 have been shown to be involved in the species barrier.

Xenon binding site C has a large surface and comprises residues Y149, Y150, N153, R156, Y157, Q186, H187, T190, T191, K194, E196, N197, F198, D202, M205, M206. The cavity is large enough for accepting a water molecule in all structures of the two data sets. It is also large enough to accept a xenon atom in the majority of the structures given, resulting in an apparent xenon dissociation constant *K*_app_, of 11.4 mM and a corresponding free energy Δ*G*_12_ of <3.4 kJ mol^−1^. Mutations of F198, whose aromatic ring interacts directly with xenon, are implicated in the *Gerstmann-Sträussler-Scheinker* (GSS) syndrome.

The xenon binding cavity D is characterized by chemical shift changes of residues preceding the first β-strand of the prion protein (V122, G123, G126, G127, and Y128) but it is also formed by K185, Q186, V189, and T193. Structural models of the part preceeding the first β-strand are only deposited in the PDB-file 2KUN and have been omitted in 1QM1 since it does not adopt a single well defined rigid structure. However, it shows a number of intra residual NOEs as well as ^15^N-T_1_, T_2_, and ^1^H-^15^N-NOEs that are typical for regions with residual local structure^42^. Using a water molecule as probe, a cavity D is observed in 14 out of 20 structures deposited. These observations confirm the validity of the NMR structures in this region, at least as transient conformations in thermodynamic equilibrium. Peptides encompassing the region from amino acids 106 to 127 that form the xenon binding site D are also known to reproduce the main neuropathological features of prion-related transmissible spongiform encephalopathies^13^. The chemical shifts of residues inside this region (M109, M112, A113, G114) and thus their local environments are also influenced by xenon binding to cavity D. As revealed by NMR-spectroscopy in oligomers formed by reduced prion protein the AGAAAAGA motif (amino acid 113 to 120) is strongly immobilized^20^. This palindromic motif is required for oligomerisation and the fibril formation of PrP^19^,^47^,^48^.

### Correlation between high pressure response and xenon binding sites

It is known that large high-pressure effects are often observable close to cavities in proteins indicating local conformational transitions. Since xenon binding to cavities is also accompanied by local conformational transitions the two effects should be correlated. Table 1 and Fig. 5 show that indeed the two effects correlate quite well supporting the idea that xenon binding as well as pressure response is due to structural transitions.

### Structure of the N-terminal region

In monomeric prion protein the N-terminal part appears to be mainly unstructured and from the NMR point of view rather mobile although high pressure NMR reveals some residual structure^26^. Even after oligomerisation formed by reduced prion protein the NMR spectra of seven tryptophan residues contained in the N-terminal part of the prion protein (amino acid 31 to 99) indicate a high peptide-like mobility^20^. An exception seems to be the supposed copper binding octarepeat (amino acids 60 to 91) in the presence of Cu^2+^- or Zn^2+^-ions that might have a physiological role in neuroprotection^35^,^36^,^37^. In the absence of metal-ions DEER-experiments with spin labels at position of Q61 and T200 of mouse PrP show that the distance distribution of the spin labels is characteristic for a conformational equilibrium where the octarepeat region is close to the core domain in about 20% of the structures. Zn^2+^-binding to the octarepeat induces an almost complete shift of conformational equilibrium to a state where the octarepeat region is close to T200 at the start of helix 3. It leads to chemical shift and line widths changes at M128 and in the regions encompassing F140 to Y145 in loop 1, Q171 to T182 at the start of helix 2, and K203 to Y217 in helix 3^41^. These chemical shift changes differ from the chemical shift changes observed upon removal of the N-terminal segment, where residues 187 to 193 located in the C-terminal part of helix 2 and residues 219 to 226 in the C-terminal part of helix are affected^8^. A somewhat better correlation is found for the residues identified by high pressure NMR spectroscopy: significant differences in the NMR detected pressure response after removal of the N-terminus^26^ were observed for G127, E168, H187, T192, E207, E211, and Y226.

Unfortunately, only a few residues of the octarepeat region itself could be resolved with sufficient accuracy in the xenon experiments. They do not show a specific xenon response.

The deimination of R27, R40 and R51 in recombinant PrP caused an alteration of the copper-binding affinity of the octarepeats^49^ although it is rather far away from the metal binding site. In this region K24, R25, and N47 show a significant effect upon xenon binding, probably by perturbing the transient interactions with the core domain by e.g. stabilizing cavity D.

For further analysis of interdomain contacts nitroxide spin labels based on disulphide and maleimide linking chemistry were applied and results were compared to previously published data as well as xenon binding experiments reported here. In principle, a reduction of the cross peak volumes should occur when the spin label is close to a nucleus under consideration. Such a cross peak volume reduction is observed for many residues of PrP (Fig. 6). However, some residues show negative relative changes of signal volumes most likely arising from remaining paramagnetic label in solution, resulting from incomplete washing after the coupling reaction. Low concentrations of paramagnetic agent have shown to enhance relaxation properties^43^. Since quantitative analysis of paramagnetic relaxation experiments in a multistate equilibrium predicted for the N-terminal segment are very error-prone we only qualitatively account stretches of residues showing continuous paramagnetic effects.

In general, more or less pronounced paramagnetic effects of different strength are observed all over the protein sequence (23–231) in both variants, meaning that the flexible spin-labeled N-terminal tail transiently contacts the globular domain in different locations as predicted by the DEER experiments^41^. The two spin labels were placed at two strategically interesting positions, position 93 is close to the octarepeat region extending from amino acid 60 to 91 and position 107 is located in front of the palindromic motif AGAAAAGA (residues 113–120). Effects of the spin labels in positions 93 and 107 on distant N-terminal amino acids as well as non-uniformly expressed paramagnetic effects suggest a transiently ordered conformation of this part of the sequence. Due to the lack of experimental NOE distance restraints in the flexible tail no structural data is available. The high flexibility and the repetitive nature of the octarepeat region prevented resonance assignments and through-space structural information. Few remaining signals, however, show strong paramagnetic influence especially in G93C, meaning that residue positions 50–81 are in close spatial proximity to the spin label at position 93 (Fig. 6, Table S2). G93, W99, K101, S103, K106, M109, K110, M112 show a significant non-random pressure response^26^. In this region also significant effects downstream from the positions of the spin labels are observed. Surprisingly, the N-terminal AGAAAAGA (113–120) motif is affected several times weaker by the spin label at position 93 than preceding or successive parts of the sequence.

In spin labelled G93C and T107C many residues in helix 2 and 3 of the globular domain show significant paramagnetic influence with the strongest effects on the C-terminal end of helix 3 (Fig. 6) that cannot be explained with the structure shown in Fig. 7. The spin label at position 107 shows strong effects (>2*σ*_0_) for M166 and Q172 at the start of helix 2, in the preceding loop 4, and at Y226, Q227, R228, G229 at the end of helix 3. A structure such as shown in Fig. 5 could qualitatively explain this effect. Strong paramagnetic effects in the same regions are also found for the spin label in position 93 that should also come close to these regions. Zahn *et al*.^8^ predicted as one of the possible interaction sites amino acids 219 to 226. Among others E168 and Y226 were predicted by high pressure NMR spectroscopy as possible interaction sites with the N-terminal domain. In the presence of Zn^2+^-ions the octarepeat region located close to the spin label at position 93 is predicted to also be in contact with amino acids Q171 to T182 at the start of helix 2, and K203 to Y217 in helix 3^41^. Mapping these effects on a structure of the prion protein shows that in the time-average the spin labels at position 93 and 107 and probably the whole N-terminus preferentially interacts only with one side of the protein, whereas on the opposite side there are only small effects (Fig. 7). A similar but not identical arrangement was also proposed by^41^. This is also reflected in the minimal paramagnetic effects observed in the helix 2-loop-helix 3 region on the opposite site of the globular domain. This is in agreement with the fact that in the fully matured cellular form of the prion protein, two glycans in position Asn 181 and Asn 197 are blocking any interdomain interaction.

Addition of phosphatidylglycine (POPG) and RNA leads to a conversion of recombinant PrP into a highly pathogenic protease resistant form rPrP-res similar to the pathogenic PrP^Sc^ isoform^50^. Again, the interaction affecting the properties of PrP occur to a large degree in the flexible amino terminus. Specifically, the POPG interaction takes place through the N-terminal positively charged domain and a hydrophobic domain encompassing residues 100–134^51^, which are indicated to be at least transiently close in space by the spin label experiments.

Other residues strongly influenced by the spin labels, as e. g., K186 cannot be explained by a structure such as that shown in Fig. 5. This is also true for many residues predicted as possible interaction sites of the N-terminus. However, the position of the first stretch of amino acids is not defined by NOEs in the original structure. A structure where the N-terminal domain is directly in contact with the globular domain (as e.g. shown in Fig. 5) can only represent a weakly populated structural state in the equilibrium, since otherwise homonuclear NOE data could be observed leading to a defined structure of the N-terminal domain. When this state is associated with the formation of the xenon binding cavity D, an estimate of its relative population would be 0.25 (Table 2), which agrees well with the DEER-data predicting similar populations in the absence of Zn^2+^-ions. In agreement with the assumption of multiple states of the N-terminal region molecular dynamics calculations using the PRE effects as restraints could not find a unique structure explaining all restraints.

In the set of NMR structures given of huPrP(Q212P) by Ilc *et al*.^42^ the orientations of the N-terminal region (90–110) relatively to the folded core are not unique. However, the set contains several structures that could explain our experimental data (existence of cavity D as well as paramagnetic effects between the N-terminal domain and the globular domain) and that are shown in Figs 2 and 5. In this model the loop containing the spin label at amino acid 107 is fixed by two interactions to the core: K104 could interact with Y225 and K110 could form a salt bridge with E168 or a hydrogen bond with S170 (Fig. 5). These relations are shown in more detail in Fig. S1 and are in qualitative agreement with the paramagnetic data (Table S2). K104, E168 and E211 (in blue) display significant high pressure effects as well as effects from xenon binding. The positively charged side chain of K110 exhibits a distance of 0.47 nm to the hydroxyl group of S170 and of 0.56 nm to the carboxyl group of E168. The amino group of K104 is separated by 5.9 Å from the hydroxyl group of Y225.

### Conformational states of PrP and cavity fluctuations

Four different states of Syrian hamster PrP^C ^15 as well as human PrP^C ^26 were stabilized by high pressure and characterized by high resolution multidimensional NMR at atomic detail. Moreover, species-specific differences in the response upon hydrostatic pressure were observed by high-resolution multidimensional NMR spectroscopy for the core domain (121–230) of Syrian hamster and human PrP^C ^15. Both proteins fluctuate between two well-folded conformations N_1_ and N_2_ and two excited states I_1_ and I_2_^15^. Although the amino acid sequence between both prion proteins is nearly identical (only 14 amino acids out of 110 residues are different between both core domains) Kremer *et al*.^15^ observed significant differences of the pressure response for the individual residues. This indicates that the secondary structure of the complete folded core, which is nearly identical for all mammalian PrP proteins, is affected differently by high hydrostatic pressure implying structurally different excited intermediates. Oligomers formed by reduced human prion *hu*PrP^C^(23–231) dissociate under pressure into monomers that occur in rare, metastable excited states of PrP^C^ stabilized at high pressure^20^.

In the full-length *hu*PrP^C^(23–230) residues K101, S103, K104, K106, N108, M109, and M112 of the unfolded amino-terminal region showed significant chemical shift changes upon application of high pressure. In the structured core domain of *hu*PrP^C^ significant pressure shifts of residues M129, G131, I139, H140, F141, C179, T183, Q186, H187, T188, V189, T191, K194, G195, E196, N197, F198, T199, D202, M205, E207, V210, E211, E219, and R220 are observed. Most of them are close to the five cavities (xenon binding sites) described here (Table 1, Figs 2 and 5).

The NMR structures as well as the xenon binding data indicate that these cavities are in a dynamic equilibrium between open and closed states. Such an equilibrium between conformational states where the cavities are open and contain water or where they are closed and too small to accept a water molecule could explain the observed pressure effects: The free energy difference Δ*G*^0^_12_ between the two native states *N*_1_ and *N*_2_ is 3.0 ± 1.4 kJ mol^−1^ and the volume difference Δ*V*^0^_12_ is −86 ± 17 mL mol^−1^ (0.14 ± 0.03 nm^3^)^15^. A positive Δ*G* combined with negative Δ*V* value means that the state dominating at ambient pressure gets rare at high pressure and vice versa. Cavities A_1_, A_2_, B, C, and D are characterized by free energy differences between the water containing and the closed state of 3.8, 4.7, 2.1, >9.0, and 2.7 kJ mol^−1^, respectively. For all cavities except cavity C these free energy values satisfactorily agree with the energy difference between state *N*_1_ and *N*_2_. However, according to the definition of Δ*G*_***H2O***_given in Table 2 all cavities preferentially contain at least one water molecule at ambient pressure. If the transition observed by high pressure NMR would describe the equilibrium between an open state containing a water molecule and a closed state excluding water, then at low pressure the water containing state *N*_1_ should prevail but would be replaced by closed state *N*_2_ at high pressures. It is usually observed that high pressure favours an increase of the size of hydrated cavities, thus the structural states derived from the pressure response do probably not describe the hydration of the cavity. In the present case it would lead to a further hydration (opening) of the cavities, as it is required for xenon binding. If the process described by the high-pressure experiments would describe the opening of a cavity to a size large enough to accommodate a xenon atom then cavity A2 and cavity D would be appropriate candidates with Δ*G*_12_^0^ values of −4.2 and −3.7 kJ mol^−1^, respectively. Both are located close to an area probably involved in the fibril formation process. The observed volume difference would correspond to the volume of approximately 5 water molecules with a molar volume of 18 mL mol^−1^. The van-der-Waals volume of a xenon atom calculated from a radius of 0.22 nm would be 27 mL mol^−1^, which is the total volume difference that would correspond to approximately three xenon atoms bound.

The free energy differences for the transition from the native state to intermediate states *I*_1_ and *I*_2_ are 10.8 ± 1.9 kJ mol^−1^ and 18.6 ± 2.9 kJ mol^−1^, respectively^15^,^26^, the corresponding volume differences are −66 ± 26 mL mol^−1^ and −125 ± 32 mL mol^−1^. Since the Δ*G*^0^-values were mainly determined from cross peak volume changes, the sign of the free energy difference is uniquely defined. The large Δ*G*^0^ values indicate that the states assumed at high pressure are dominant at ambient pressure. This means that the enlargement of the cavities cannot be correlated with the transition to the intermediate states but has to describe another process on the pathway to PrP^Scr^.

## Conclusion

We have shown here that by use of xenon as a probe it is possible to detect cavities in proteins that in the deposited structures are too small to accept a xenon atom (e.g. cavity B) but have to be transiently enlarged. We have derived here the corresponding equations (see Materials and Methods) for a two state model with an open and a closed state of cavities in equilibrium. The functional dependence is different from a simple one state binding model, since the data can only be satisfactorily fitted by a model allowing more than one state. Qualitatively, such an effect is experimentally observed here (Fig. 4). However, for a quantitative description the number of parameters to fit was too high, thus we assumed that the NMR data reflect the ratio of opening and closing as a first approximation. In principle, using more data points (which are not easy to obtain) it should be possible to completely extract all parameters with sufficient accuracy.

The N-terminal region is clearly not in a uniquely folded structure but nevertheless xenon effects are observable in a well-defined region, which is populated for at least a fraction of time. Here, xenon is able to detect a transient binding site (cavity D) that is reported in some of the NMR structures of the structural ensemble if 2KUN where the partly folded N-terminal region is in contact with the core region.

High pressure NMR is also known to detect fluctuations close to cavities and thus indirectly cavities. In fact, we can observe a good correspondence between our xenon data set and the high pressure data published before. In general, transient structures in a multistate equilibrium as derived from NMR-parameters of the N-terminal domain typical for a disordered and highly mobile protein segment and partly quantified by DEER experiments^41^ cannot be determined exactly by experiments only, but experimental data can be used to select or discard models as we have done here. Another supplementary method is the use of spin labels that can detect transient interaction sites but again cannot be used for a direct structure determination when no unique structure exists (as in our case).

From the biological point of view fluctuating cavities are structurally weak points where conformational transitions are often initiated and which we have now characterized for the prion protein. Cavities A2 is formed in part by the loop between β-strand 2 and α-helix 2 from residue 166 to 172, a region that is highly important for species differences in infectivity and that is involved in a conformational transition between a 3_10_-helix in one state, and a type-I β-turn in the other state. In most structures it is too small to contain a xenon atom and has therefore to open up in a dynamic equilibrium. Cavity D is located in a region that is supposed to be mandatory for fibril formation, xenon binding induces chemical shift changes in the range from M112 to H155, a stretch of amino acids that contains the AGAAAAGA (113–120) motif and the first β-strand. Xenon NMR shows that at least transiently this part of the N-terminal segment is in close contact with the folded core. The *N*_1_ to *N*_2_ transition observed earlier by high pressure NMR spectroscopy could describe the opening of these two cavities for an atom of the size of a xenon atom. The combined high pressure and spin label study clearly demonstrates that the N-terminal segment has at least transient contacts with the back side of the folded core of the prion protein, being in agreement with some of the NMR structures deposited in the 2KUN ensemble of structures. This may also be the natural position when the prion protein is bound to the membrane.

The UniProt data base (see “ http://www.uniprot.org/uniprot”) lists 17 positions in the folded core of human PrP that are correlated with the development of FFI, CJD, or GSD. Although these mutations do not occur more frequently in residues located at the surface of cavities, nevertheless they may influence the dynamic stability of the cavities. However, here experimental data are missing. A surprisingly high correlation is found between these critical locations and residues that show significant chemical shift changes with pressure: 16 of the 17 pathogenic mutations in the core domain are located in pressure sensitive positions or are direct neighbours of one of the 25 pressure sensitive positions. This again would be in a good agreement with the assumption that the pressure sensitive residues identify structurally weak points of the structure that are prone to trigger a transition to a pathogenic state.

## Materials and Methods

### Site directed mutagenesis, protein production, and sample preparation

The tag-less expression construct for prion protein huPrP(23–231) in pET-17b vector was a kind gift from Dr. Uwe Bertsch. PrP-variants G93C and T107C were generated by site-directed mutagenesis following the Quick-Change protocol (Promega). The results of mutagenesis were checked by sequencing (AGOWA GmbH, Germany). For protein production fresh transformations of each construct in *Escherichia coli* BL21 (DE3) pLysS (Novagen, Merck KGaA, Darmstadt, Germany) were prepared by electroporation (MicroPulser, Biorad, USA). Cells were grown in lysogeny broth medium and were subsequently transferred to 1 L of ^15^N-isotopically labelled M9 medium. Overexpression was induced at an OD_600_ of 0.7 by the addition of 1 mM isopropyl-β-D-galactopyranoside (IPTG) and inclusion body formation was enhanced by continuous expression at 42 °C. The cells were harvested 4 h after induction by centrifugation at 4 °C and stored at −20 °C until further processing. Purification of PrP-inclusion bodies was performed as described by Eberl *et al*.^52^. Subsequently, refolding was achieved on a Ni-NTA column (Qiagen N. V, Germany) by exploiting the protein’s intrinsic metal-binding sites. Fractions were checked by SDS-PAGE and pure fractions were pooled. The protein was dialysed against ddH_2_O with 0.02% acetic acid, pH 3.5, freeze-dryed (Christ alpha 1–4, Sartorius BBI Systems GmbH, Germany), and stored at −20 °C for further analysis.

### Spin labeling

For site-directed spin labeling 1-oxyl-2,2,5,5-tetramethyl-∆3-pyrroline-3-methyl methanethiosulfonate (MTSL) and 1-oxyl-3-(maleimidomethyl)-2,2,5,5-tetramethyl-1-pyrrolidine (Toronto Research Chemicals, Canada) for G93C and T107C, respectively, were dissolved in acetonitrile and dimethyl sulfoxide, respectively, to final concentrations of 10 mg/ml. Approximately 5 mg of freeze-dried full-length PrP variant protein were dissolved in 10 mM sodium acetate buffer, pH 4.5 containing 5 mM dithiothreitol (DTT) and shaken for 1 h before excess reducing agent was removed by buffer exchange using a Vivaspin filtration unit (cutoff 10 kDa, Merck Millipore, Germany). The concentration of the protein was monitored using the absorbance at 280 nm (*ε*_280nm_ = 57995 M^−1^cm^−1^) until a concentration of at least 200 μM was reached. 50 μl of the respective spin label reagent corresponding to 10- to 20-fold molar excess were added immediately, mixed, and incubated over night at room temperature. Excess spin label was removed by buffer exchange as described above. The sample was concentrated to about 600 μl followed by addition of 66 μl (10% v/v) of D_2_O as a NMR lock substance. Prior to NMR measurements the pH was readjusted to 4.5. Diamagnetic conditions were achieved by adding 3.3 μl of freshly prepared 5 mM ascorbic acid in 10 mM sodium acetate buffer (pH 4.5) to the sample. Dilution of the sample was negligible (0.5% v/v). [^1^H,^15^N]-HSQC spectra were recorded on a Bruker Avance 700 NMR spectrometer equipped with a 5 mm TCI cryogenic probe at 298 K. The ^1^H and ^15^N spectral widths were 9765.6 Hz and 1845 Hz, respectively. 1024 × 256 data points were recorded for each spectrum.

### ^1^H-^15^N correlation spectroscopy in the presence of ^129^Xe

The NMR samples used for the xenon experiments contained 0.25 mM of ^15^N-enriched huPrP(23–230) in 5 mM acetate buffer, 0.1 mM of 2,2-dimethyl-2-silapentane-5-sulfonate (DSS), pH 4.5 in 90% ^1^H_2_O/10% ^2^H_2_O. All NMR experiments were recorded at 293 K. Xenon-loaded samples were measured on a Bruker Avance 500 operating at a ^1^H resonance frequency of 500.12 MHz. Sensitivity enhanced [^1^H,^15^N]-HSQC spectra^53^,^54^,^55^ were recorded with echo-antiecho detection (Bruker pulse program hsqcetf3gpsi). The ^1^H and ^15^N spectral widths were 8012 Hz and 1825 Hz, respectively, recording 2048 × 512 data points. DSS was used as internal reference for ^1^H chemical shifts, ^15^N shifts were referenced indirectly to DSS using a Ξ-factor of 0.101329118^56^. Spectral assignment was based on the data published by Zahn *et al*.^8^ as adapted by Kachel *et al*.^26^. The xenon concentration in the aqueous solution is calculated using a solubility coefficient of 44 mM MPa^−1^ from the xenon partial pressure in the NMR tube^28^. Samples were measured in the absence of xenon and at xenon pressures of 0.2, 0.4, 0.8 and 1.4 MPa.

### Xenon high-pressure equipment

Samples were pressurized using a homemade high-pressure apparatus equipped with a sapphire tube (Saphikon, USA) similar to the one used by Baumer *et al*.^57^. Samples were filled with natural abundance xenon gas (Xenon 4.0 > 99.99%, Linde Gas AG, Germany).

### Data processing

Spectra were processed with the Topspin package (Bruker BioSpin) or in-house software and further analysed with AUREMOL^58^ by automatic module to obtain the volume and chemical shift changes or the CcpNmr software package^59^.

### Cavity calculation

Cavity volumes and solvent accessible surface were calculated using the web servers CASTp^60^ and POCASA^61^, and also with the programs MOLMOL^62^ and PyMol^63^. The cavity volumes given in Table 1 were obtained by CASTp. The PDB structures 1QM2 and 2KUN were used for the cavity determination. Probe radii of 1.2 Å (water) and 2.2 Å (xenon) were used. PyMol was used for the visualization and analysis of data. The results from CASTp only are shown.

## Modeling of Xe-bound huPrP

The structure of Xe-bound huPrP was modeled using CNS 1.21 program suite^64^. The PDB structure 2KUN (90–237) Q212P was used as the initial structure for modeling Xe-bound *hu*PrP. The xenon binding positions were chosen based on the NMR data and cavity positions identified. Pseudo distance restraints were generated for xenon positioning for all residues at the surface of a cavity that show chemical shift and/or cross peak volume changes upon xenon binding in [^1^H,^15^N]-HSQC spectra. An upper distance limit for the xenon-proton distances of 0.54 nm and a lower distance limit of 0.34 nm was selected. Modeling of the (90–230) Q212 fragment of *hu*PrP was done using the substitute restraint method as described by Cano *et al*.^65^ along with the pseudo distance restraints generated for Xenon atoms positioned into the cavities. 7365 substitute restraints (6720 distances, 587 dihedral angles and 58 H-bonds) were generated by PERMOL^66^ using the PDB structure 2KUN and replaced the primary experimental restraints as specified by Cano *et al*.^65^. No restraints were applied to the side-chains of the residues around the Xe-binding sites, allowing free movements of the side-chains. Simulated annealing was done with the simulated annealing protocol of CNS employing substitute restraints to the initial extended structure and also the pseudo distance restraints generated for xenon atoms. Cartesian dynamics was applied. A refinement was also carried out in explicit water. 100 structures were generated and the lowest energy structure (−17 829 kJ mol^−1^) with optimum geometry was selected for further analysis. PyMol^63^ was used for the visualization and analysis of modeled Xe-bound *hu*PrP structure.

### Evaluation of the chemical shift and volume changes induced by xenon binding

Combined amino acid specific chemical shift differences Δ*δ*_comb_ of the amino acids *j* were calculated according to Schumann *et al*.^67^ by


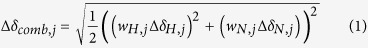


where Δ*δ*_*H,j*_ and Δ*δ*_*N,j*_ are the chemical shift differences of the amide proton and nitrogen of amino acid *j* in the partly complexed and free protein, respectively. The weighting factors *w*_H,j_ and *w*_N,j_ are specific for the kind of atom and the type of amino acid in position *j*. The chemical shift changes Δ*δ*_comb,j_ were considered as significant when they were larger than the corrected standard deviation σ_0_^corr^ to zero.

For *N* independent binding sites *i* with the microscopic association constants *k*^i^ the occupancy 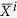
of the site *i* can be calculated from the microscopic association constants *k*^i^ by


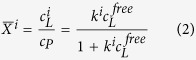


with *c*_L_^i^ the concentration of the ligand bound to site *i* and *c*_L_^free^ the concentration of the free ligand and *c*_P_ the total protein concentration^68^.

When the chemical shifts at site *i* are not significantly influenced by the binding site *j (j*≠*i*) and fast exchange conditions prevail, the occupancy of site *i* can be obtained from


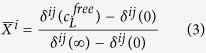


with *δ*^ij^(c_L_^free^) the chemical shift of residue *j* in site *i* at a given ligand concentration of the free ligand.

From equations (2) and (3) follows that


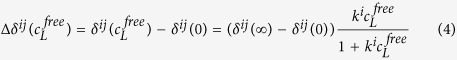


Under slow exchange conditions the association constant *k*^i^ can be calculated as


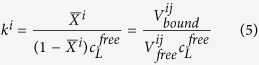


or


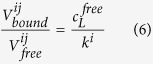


with *V*^*ij*^_*free*_ and *V*^*ij*^_*bound*_ the cross peak volumes of a resonance *j* in the ligand free protein and in the protein with the ligand *L* bound. Again it is assumed that resonance *j* only senses the binding to site *i*. When the total protein concentration is constant and slow exchange conditions prevails for the transition but the cross peak of the bound state could not be identified, equation (7) applies.


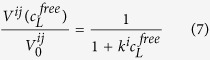


with *V*^*ij*^_*0*_ = *V*^*ij*^_*free*_ + *V*^*ij*^_*bound*_ = *V*^*ij*^(*c*_*L*_^*free*^ = 0)

When the protein exists in two states 1 and 2 in equilibrium with different binding constants *k*_*1*_^*i*^ and *k*_*2*_^*i*^ for the ligand L, the observed volume changes depend on the time scales of the four different transitions (Fig. 3). The equilibrium between the two conformational states of the protein can be described by the constants *k*_*3*_^*i*^ = [1]/[2] and *k*_*4*_^*i*^ = [3]/[4]. If there is slow exchange for the transition between the two states of the protein and fast exchange for ligand binding, the cross peak volume of site i in the conformational state 1 is


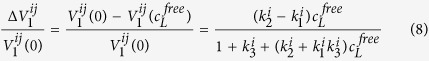


When the cavity can bind xenon in state 2 only, because xenon is too large to fit into the cavity in state 1 (*k*_*1*_^*i*^ = 0) eq. 8 simplifies to


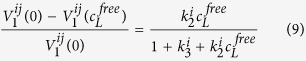


If fast exchange conditions apply for all states, the chemical shift *δ*(c_L_) is given by


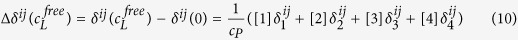


The concentrations of the different states can be calculated as


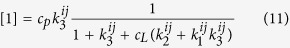



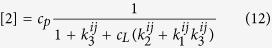



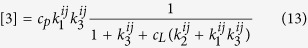



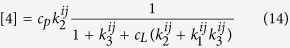


Substituting eq. 11 into eq. 10 gives the general solution


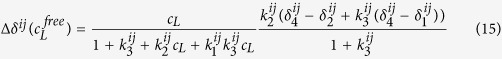


For *k*_1_ = 0 one obtains


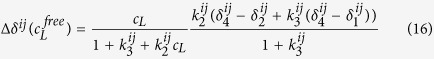


## Additional Information

**How to cite this article**: Narayanan, S. P. *et al*. Structural transitions in full-length human prion protein detected by xenon as probe and spin labeling of the N-terminal domain. *Sci. Rep.* **6**, 28419; doi: 10.1038/srep28419 (2016).

## Supplementary Material

Supplementary Information

## Figures and Tables

**Table 1:** Residues influenced by xenon binding^a^.

Residues	Location
K24, R25, *N47, **M109***, **M112**	unfolded N-terminus
A113, *G114*, A117	AGAAAAGA motif
V122, G123, G126 G127, Y128	loop 0
**G131**	β-strand 1
*S132*, M134, *S135*, **R136**, *I138*, **I139, H140**, **F141**, *G142*	1oop 1
Y145, ***Y146***, D147, *Y149,Y150*, **R151**, *E152*	α-helix 1
***N153***	loop 2
H155, *R156*	3/10 helix 1
*Y157*	loop 3
Y162	β-strand 2
M166, E168, ***S170***	loop 4
*N173, **N174**, V176, D178, C179*, V180, *N181, **T183***, **I184**, ***T188**,*	α-helix 2
K194	3/10 helix 2
G195, **F198**	loop 5
**E200**, *M205*, M206, *R208*, V209, E211, *Q212, **I215***, T216, ***Q217***, E219	α-helix 3
**E221**	loop 6
*S222, A224,* G229	α-helix 4

^a^Residues showing significant (≥*σ*_0_) chemical shift or cross peak volume changes with xenon binding are depicted in normal letters and italics, respectively. Residues that have at least one pressure coefficient *B*_1,2_ > 2 *σ*_0_^15^,^22^ are depicted in bold letters.

**Table 2:** Cavities and xenon binding sites in the human PrP^a^.

Binding Site	Cavities/pockets in 3D structures				
	Surrounding residues^b^	PrP(90–237) (2KUN)		PrP(90–230) (1QM1)	
	0.12 nm probe	0.12 nm probe^c^	0.22 nm probe^c^	0.12 nm probe^c^	0.22 nm probe^c^
		Volume [nm^3^]			
A_1_	**G131**, **S132**, **M134**, **V161**, **Y162**, Y163, F175, **V176**, **C179**, C214, **I215**, **Q217**, Y218, **E219**, R220	0.058 (0.023) 18/20	0.002 (0.007) 1/20	0.045 (0.037) 15/20	0.0 0/20
A_2_	R164, **E168**, **S170**, N171, **N173, N174**	0.041 (0.025) 19/20	0.005 (0.017) 2/20	0.049 (0.039) 16/20	0.011 (0.024) 4/20
B	**I138**, **I139**, **F141**, **E146**, **D147**, **Y150**, **Y157**, **M205**, **R208**, **Y209**	0.017 (0.025) 8/20	0.0 0/20	0.069 (0.019) 20/20	0.0 0/20
C	**Y149**, **Y150**, **N153**, **R156**, **Y157**, Q186, H187, T190, T191, **K194**, E196, N197, **F198**, D202, **M205**, **M206**	0.144 (0.049) 20/20	0.080 (0.040) 18/20	0.146 (0.075) 20/20	0.042 (0.035) 14/20
D	A120, V121, **V122, G123**, G124, L125, **Y128**, **Y162**, K185, Q186, V189, T193	0.081 (0.114) 14/20	0.044 (0.090) 5/20	–d	–d

^a^Cavities were calculated with CASTp using two different probe radii (water, 0.12 nm; Xe, 0.22 nm). The structural ensembles of the PDB entries 2KUN and 1QM1 have been used for the analysis.

^b^Residues contributing at least one atom to the surface of the cavities determined with a sphere of 0.12 nm radius in the 20^th^ structure of the ensembles of the PDB entry 2KUN and the 1^st^ structure of the PDB ensemble 1QM1, respectively. Residues in bold letters show significant xenon effects above *σ*_0_.

^c^The cavities were calculated for all 40 structures of the two PDB data sets deposited. The mean volume and the standard deviation of the mean value (values in brackets) are listed for the cavities defined by a probe of the size of a water molecule and a xenon atom, respectively. In addition, the number of structures is listed where a cavity can be detected that is large enough for accepting a water molecule or a xenon atom.

^d^N-terminal part is not contained in the PDB entry 1QM1.

**Table 3:** Equilibrium constants and free energies for xenon binding to *hu*PrP (23–230)^a^.

Binding Site	*k*_2_[mM^−1^]	Δ*G*_*24*_[kJ M^−1^]	*p*_open_^b^ Xe	*k*_3_^b^ Xe	*K*_app_ Xe [mM]	Δ*G*_*12*_[kJ M^−1^]	*p*_open_^b^ H_2_O	Δ*G*_*H2O*_[kJ M^−1^]
A_1_	3.2 ± 0.4	−19.7 ± 0.3	0.025	39	12.5	−8.9	0.83	3.8
A_2_	0.46 ± 0.01	−14.91 ± 0.03	0.15	5.7	14.6	−4.2	0.88	4.7
B	4.5 ± 0.4	−20.5 ± 0.2	<0.024	>39^c^	9.1	<−9.0	0.7	2.1
C	0.11 ± 0.03	−11.5 ± 0.7	0.80	0.25	11.4	3.4	>0.98	>9.0
D	0.34 ± 0.02	−14.24 ± 0.02	0.25	3.0	11.8	−2.7	0.7	2.7

^a^Mean association constants *k*_j_^i^ for binding sites i were calculated with eqs 9 and 16 assuming *k*_1_ = 0. Temperature 293 K. For definition of the constants see Fig. 3.

^b^*p*_open_, probability of the open state in absence of xenon calculated from the NMR structures 2KUN and 1QM1 (Table 1). Site D is only transiently formed by N-terminus and thus the state, where the cavity is formed corresponds to the open (binding) state. *k*_3_ has been calculated from *p*_open_ as *k*_3_ = (1 − *p*_open_)/*p*_open_. The apparent xenon dissociation constant *K*_app_ is defined by *K*_app_ = 1/*k*_2_ + *k*_3_/*k*_2_.

^c^In none of the NMR structures cavity B is large enough for accommodating a sphere of the size of the xenon atom. However, a smaller population than 0.025 is not excluded by statistics. For the calculations the constant *k*_2_ was set to 39.

## References

[b1] Prusiner S. B. Prions. Proc. Natl. Acad. Scl. USA. 95, 13363–83 (1998). 10.1073/pnas.95.23.13363 PMC33918 9811807

[b2] Knight R. Creutzfeldt-Jakob Disease: A Rare Cause of Dementia in Elderly Persons, Clin Infect Dis. 43, 340–346 (2006). 16804850 10.1086/505215

[b3] Brown P., Brandel J. P., Presse M. & Sato T. Iatrogenic Creutzfeldt - Jakob disease: The waning of an area. Neurolocy. 67, 389–393 (2006). 10.1212/01.wnl.0000231528.65069.3f 16855204

[b4] Brown K. & Mastrianni J. A. The Prion Diseases. J Geriatr Psychiatry Neurol. 23, 277–298 (2010). 20938044 10.1177/0891988710383576

[b5] Norby E. Prion and protein-folding diseases (Review). J Intern Med. 270, 1–14 (2011). 21481020 10.1111/j.1365-2796.2011.02387.x

[b6] Prusiner S. B. Novel proteinaceous infectious particles cause scrapie. Science. 216, 136–44 (1982). 6801762 10.1126/science.6801762

[b7] Soto C. & Catilha J. The controversial protein-only hypothesis of prion propagation. Nat Med. 10, 63–67 (2004). 10.1038/nm1069 15272271

[b8] Zahn R. . NMR solution structure of the human prion protein. Proc. Natl. Acad. Scl. USA. 97, 145–150 (2000). 10.1073/pnas.97.1.145 PMC26630 10618385

[b9] Herrmann L. M. & Caughey B. The importance of the disulfide bond in prion protein conversion. Neuro. Rep. 9, 2457–2461 (1998). 10.1097/00001756-199808030-00006 9721914

[b10] Tabrett C. A. . Changing the solvent accessibility of the prion protein disulfide bond markedly influences its trafficking and effect on cell function. Biochem. J. 428, 169–182 (2010). 20337594 10.1042/BJ20091635

[b11] Wickner R. B., Edskes H. K., Shewmaker F., Kryndushkin D. & Nemecek J. Prion variants, species barriers, generation and propagation. J. Biol. 8, 47–51 (2009). 19519931 10.1186/jbiol148 PMC2736662

[b12] Ziegler J. . CD and NMR Studies of Prion Protein (PrP) Helix 1. J. Biol. Chem. 278, 50175–81 (2003). 12952977 10.1074/jbc.M305234200

[b13] Kuwata K. . NMR-detected hydrogen exchange and molecular dynamics simulations provide structural insight into fibril formation of prion protein fragment 106–126. Proc Natl Acad Sci USA 282, 14790–95 (2003). 14657385 10.1073/pnas.2433563100 PMC299804

[b14] Kuwata K. . Hot spots in prion protein for pathogenic conversion. Proc. Natl. Acad. Sci. USA 104, 11921–26 (2007). 17616582 10.1073/pnas.0702671104 PMC1924567

[b15] Kremer W., Kachel N., Akasaka K. & Kalbitzer H. R. Species-specific Differences in the intermediate States of Human and Syrian Hamster Prion protein detected by high pressure NMR spectroscopy. J Biol Chem. 282, 22689–98 (2007). 17519231 10.1074/jbc.M701884200

[b16] Henriques S. T., Pattenden L. K., Aguilar M. I. & Castanho M. A. R. B. The toxicity of Prion protein fragment PrP(106-126) is not mediated by membrane permeabilization as shown by a M112W substitution. Biochemistry. 48, 4198–4208 (2009). 19301918 10.1021/bi900009d

[b17] Hosszu L. L. P. . Conformational Properties of β-PrP. J Biol Chem. 284, 21981–90 (2009). 19369250 10.1074/jbc.M809173200 PMC2755922

[b18] Damberger F. F., Chisten B., Pérez D., Hornemann S. & Wüthrich K. Cellular prion protein conformation and function. Proc Natl Acad Sci USA. 108, 17308–13 (2011). 21987789 10.1073/pnas.1106325108 PMC3198368

[b19] Lührs T., Zahn R. & Wüthrich K. Amyloid formation by recombinant full-length prion proteins in phospholipid bicelle solutions. J Mol Biol. 357, 833–841 (2006). 16466741 10.1016/j.jmb.2006.01.016

[b20] Sasaki K. . Reversible monomer-oligomer transition in human prion protein. Prion. 2, 118–122 (2008). 19158507 10.4161/pri.2.3.7148 PMC2634530

[b21] Asante E. A. . A naturally occurring variant of the human prion protein completely prevents prion disease. Nature. 522, 478–481 (2015). 26061765 10.1038/nature14510 PMC4486072

[b22] Kalbitzer H. R. . Intrinsic Allosteric Inhibition of Signaling Proteins by Targeting Rare Interaction States Detected by High-Pressure NMR Spectroscopy. Angew. Chem. Int. Ed. 52, 14242 –14246 (2013). 10.1002/anie.201305741 24218090

[b23] Akasaka K. Probing Conformational Fluctuation of Proteins by Pressure Perturbation. Chem. Rev. 106, 1814–1835 (2006). 16683756 10.1021/cr040440z

[b24] Akasaka K. & Matsuki H. High Pressure Bioscience - Basic Concepts, Applications and Frontiers. Springer, Heidelberg, Germany (2015).

[b25] Kuwata K. . Locally disordered conformer of the hamster prion protein: a crucial intermediate to PrPSc? Biochemistry 41, 12277–12283 (2002). 12369815 10.1021/bi026129y

[b26] Kachel N., Kremer W., Zahn R. & Kalbitzer H. R. Observation of intermediate states of the human prion protein by high pressure NMR spectroscopy. BMC Struct. Biol. 6, 16–34 (2006). 16846506 10.1186/1472-6807-6-16 PMC1557509

[b27] Tilton R. F. Jr., Kuntz I. D. Jr. & Petsko G. A. Cavities in proteins: structure of a metmyoglobin-xenon complex solved to 1.9 A. Biochemistry 23, 2849–2857 (1984). 6466620 10.1021/bi00308a002

[b28] Locci E. . Probing Proteins in Solution by ^129^Xe NMR Spectroscopy. J. Magn. Reson. 150, 167–174 (2001). 11384176 10.1006/jmre.2001.2325

[b29] Rubin S. M., Spence M. M., Pines A. & Wemmer D. E. Characterization of the Effects of Nonspecific Xenon-Protein Interactions on ^129^Xe Chemical Shifts in Aqueous Solution: Further Development of Xenon as a Biomolecular Probe. J Magn Reson 152, 79–86 (2001). 11531366 10.1006/jmre.2001.2389

[b30] Rubin S. M., Lee S. Y., Ruiz E. J., Pines A. & Wemmer D. E. Detection and characterization of xenon-binding sites in proteins by ^129^Xe NMR spectroscopy. J Mol Biol. 322, 425–40 (2002). 12217701 10.1016/s0022-2836(02)00739-8

[b31] Möglich A. . Solution structure of the active-centre mutant Ile14Ala of the histidine-containing phosphocarrier protein (HPr) from *Staphylococcus carnosus*. Eur. J. Biochem. 271, 4815–4824 (2004). 15606769 10.1111/j.1432-1033.2004.04447.x

[b32] Gröger C. . NMR-spectroscopic mapping of an engineered cavity in HPr the I14A Mutant from *S. carnosus* using xenon. J. Am. Chem. Soc. 125, 8726–8727 (2003). 12862458 10.1021/ja030113t

[b33] Baumann F. . Lethal recessive myelin toxicity of prion protein lacking its central domain. EMBO J. 26, 538–547 (2007). 17245436 10.1038/sj.emboj.7601510 PMC1783444

[b34] Li A. . Neonatal lethality in transgenic mice expressing prion protein with a deletion of residues 105–125. EMBO J. 26, 548–558 (2007). 17245437 10.1038/sj.emboj.7601507 PMC1783448

[b35] Burns C. S. . Molecular features of the copper binding sites in the octarepeat domain of the prion protein. Biochemistry 41, 3991–4001 (2002). 11900542 10.1021/bi011922x PMC2905306

[b36] Millhauser G. L. Copper and the prion protein: methods, structures, function, and disease. Annu. Rev. Phys. Chem. 58, 299–320 (2007). 17076634 10.1146/annurev.physchem.58.032806.104657 PMC2904554

[b37] Walter E. D. . Copper Binding Extrinsic to the Octarepeat Region in the Prion Protein. Curr. Prot. Pept. Sci. 10, 529–535 (2009). 10.2174/138920309789352056 PMC2905140 19538144

[b38] Pauly P. C. & Harris D. A. Copper stimulates endocytosis of the prion protein. J. Biol. Chem. 273, 33107–33110 (1998). 9837873 10.1074/jbc.273.50.33107

[b39] Bocharova O. V., Breydo L., Salnikov V. V. & Baskakov I. V. Copper(II) inhibits *in vitro* conversion of prion protein into amyloid fibrils. Biochemistry 44, 6776–6787 (2005). 15865423 10.1021/bi050251q

[b40] Orem N. R., Geoghegan J. C., Deleault N. R., Kascsak R. & Supattapone S. Copper (II) ions potently inhibit purified PrPres amplification. J. Neurochem. 96, 1409–1415 (2006). 16417569 10.1111/j.1471-4159.2006.03650.x

[b41] Spevacek A. R. . Zinc Drives a Tertiary Fold in the Prion Protein with Familial Disease Mutation Sites at the Interface. Structure 21, 236–246 (2013). 23290724 10.1016/j.str.2012.12.002 PMC3570608

[b42] Ilc G. . NMR Structure of the Human Prion Protein with thePathological Q212P Mutation Reveals Unique Structural Features. PLoS One. 5, e11715 (2010). 20661422 10.1371/journal.pone.0011715 PMC2908606

[b43] Hiller S., Wider G., Etezady-Esfarjani T., Horst R. & Wuthrich K. Managing the solvent water polarization to obtain improved NMR spectra of large molecular structures. J Biomol NMR. 32, 61–70 (2005). 16041484 10.1007/s10858-005-3070-8

[b44] García F. L., Zahn R., Riek R. & Wüthrich K. NMR structure of the bovine prion protein. Proc. Natl. Acad. Sci. USA 97, 8334–8339 (2000). 10899999 10.1073/pnas.97.15.8334 PMC26948

[b45] Riek R. . NMR Structure of the mouse prion protein domain PrP(121–231). Nature (London) 382, 180–182 (1996). 8700211 10.1038/382180a0

[b46] Sigurdson C. J. . De novo generation of a transmissible spongiform encephalopathy by mouse transgenesis. Proc. Nat. Acad. Sci. USA 106, 304–309 (2009). 19073920 10.1073/pnas.0810680105 PMC2629180

[b47] Norstrom E. M. & Mastrianni J. A. The AGAAAAGA Palindrome in PrP Is Required to Generate a Productive PrPSc-PrPC Complex That Leads to Prion Propagation. J. Biol. Chem. 208, 27236–27243 (2005). 15917252 10.1074/jbc.M413441200

[b48] Abskharon R. N. N. . Probing the N-Terminal β-Sheet Conversion in the Crystal Structure of the Human Prion Protein Bound to a Nanobody. J. Am. Chem. Soc. 136, 937–944 (2014). 24400836 10.1021/ja407527p

[b49] Young D. S. . Effect of enzymatic deimination on the conformation of recombinant prion protein. Biochim. Biophys. A. 1794, 1123–1133 (2009). 10.1016/j.bbapap.2009.03.013 19341825

[b50] Wang F., Wang X., Yuan C. G. & Mal J. Generating a Prion with Bacterially Expressed Recombinant Prion Protein. Science. 327, 1132–1135 (2010). 20110469 10.1126/science.1183748 PMC2893558

[b51] Wang F. . Role of the Highly Conserved Middle Region of Prion Protein (PrP) in PrP-Lipid Interaction. Biochemistry. 49, 8169–8176 (2010). 20718504 10.1021/bi101146v PMC2950782

[b52] Eberl H., Tittmann P. & Glockshuber R. Characterization of Recombinant, Membrane-attached Full-length Prion Protein. J. Biol. Chem. 279, 25058–25965 (2004). 15031284 10.1074/jbc.M400952200

[b53] Palmer A. G. III., Cavanagh J., Wright P. E. & Rance M. Sensitivity Improvement in Proton-Detected Two-Dimensional Heteronuclear Correlation NMR Spectroscopy. J. Magn. Reson. 93, 151–170 (1991).

[b54] Kay L. E., Keifer P. & Saarinen T. Pure Absorption Gradient Enhanced Heteronuclear Single Quantum Correlation Spectroscopy with Improved Sensitivity. J. Am. Chem. Soc. 114, 10663–10665 (1992).

[b55] Schleucher J. . A general enhancement scheme in heteronuclear multidimensional NMR employing pulsed field gradients. J. Biomol. NMR. 4, 301–306 (1994). 8019138 10.1007/BF00175254

[b56] Wishart D. S. . ^1^H, ^13^C and ^15^N chemical shift referencing in biomolecular NMR. J Biomol NMR. 6, 135–140 (1995). 8589602 10.1007/BF00211777

[b57] Baumer D., Fink A. & Brunner E. Measurement of the ^129^Xe NMR chemical shift of supercritical xenon. Phys Chem Chem Phys. 217, 289–293 (2003).

[b58] Gronwald W. & Kalbitzer H. R. Automated Structure Determination of Proteins by NMR Spectroscopy. Progr. NMR Spectr. 44, 33–96 (2004).

[b59] Vranken W. F. . The CCPN Data Model for NMR Spectroscopy: Development of a Software Pipeline. Proteins 59, 687–696 (2005). 15815974 10.1002/prot.20449

[b60] Dubois L. . Probing the Hydrophobic Cavity of Lipid Transfer Protein from *Nicotiana tabacum* through Xenon-Based NMR Spectrsocopy. J Am Chem Soc. 126, 15738–46 (2004). 15571396 10.1021/ja046195i

[b61] Yu J., Zhou Y., Tanaka I. & Yao M. Roll: A new algorithm for the detection of protein pockets and cavities with a rolling probe sphere. Bioinformatics. 26, 46–52 (2010). 19846440 10.1093/bioinformatics/btp599

[b62] Koradi R., Billeter M. & Wüthrich K. MOLMOL: A program for display and analysis of macromolecular structures. J Mol Graph., 14, 51-5, 29–32 (1996). 10.1016/0263-7855(96)00009-4 8744573

[b63] Delano W. L. The PyMOL Molecular Graphics System, Version v.099rc6, Schrödinger, LLC (2002).

[b64] Brünger A. T. . Crystallography & NMR System (CNS), A new software suite for macromolecular structure determination, Acta Cryst.D. 54, 905–921 (1998). 9757107 10.1107/s0907444998003254

[b65] Cano C. . Protein structure calculation with data imputation: the use of substitute restraints. J. Biomol. NMR 45, 397–411 (2009). 19838807 10.1007/s10858-009-9379-y

[b66] Möglich A., Weinfurtner D., Maurer T., Gronwald W. & Kalbitzer H. R. A restraint molecular dynamics and simulated annealing approach for protein homology modeling utilizing mean angles. BMC Bioinform. 6, 91 (2005). 10.1186/1471-2105-6-91 PMC1127110 15819976

[b67] Schumann F. H. . Combined chemical shift changes and amino acid specific chemical shift mapping of protein-protein interactions. J Biomol NMR. 39, 275–89 (2007). 17955183 10.1007/s10858-007-9197-z

[b68] Kalbitzer H. R. & Stehlik D. On the analysis of competitive binding of various ligands to cooperative and independent binding sites of macromolecules. Z Naturforsch C. 34, 757–769 (1979). 160700 10.1515/znc-1979-9-1018

