# Supplementary material for: Structural transitions in full-length human prion protein detected by xenon as probe and spin labeling of the N-terminal domain
Source: Sci Rep. 2016 Jun 24;6:28419. doi: 10.1038/srep28419 (PMC4920026; doi:10.1038/srep28419)

Supplementary Information

Structural transitions in full-length human prion protein detected by xenon as probe and spin labelling of the N-terminal domain

Sunilkumar Puthenpurackal Narayanan[a], Divya Gopalakrishnan Nair[a], Daniel Schaal[b], Marisa B. Aguiar[a], Sabine Wenzel[b], Werner Kremer[a], Stephan Schwarzinger[b], Hans Robert Kalbitzer[a]

[a]Institute of Biophysics and Physical Biochemistry and Centre of Magnetic Resonance in Chemistry and Biomedicine (CMRCB), University of Regensburg, 93040 Regensburg, Germany.
[b]Institute of Biopolymers, NW1/BGI, University of Bayreuth, 95440 Bayreuth, Germany

Tables

Table S1 Atoms of PrP in direct van-der-Waals contact with xenon[a]

Cavity	Atoms and residues in van-der-Waals contact with xenon
A1	V161 (H^{β}, $H^{\gamma 21}$, $H^{\gamma 22}$<br>Y162 (O)<br>F175 ($H^{\delta 2}$, $H^{\varepsilon 2}$)<br>C179 (S^{γ}, H^{δ})<br>C214 (H^{α})<br>Q217 ($H^{\gamma 1}$)
A2	E168 (C^{β}, $H^{\beta 1}$, $H^{\gamma 1}$, $H^{\gamma 2}$)<br>S170 (C^{β}, $H^{\beta 2}$, H^{γ})<br>N174 (H^{α}, $H^{\beta 1}$, $H^{\beta 2}$, $H^{\delta 21}$)
B	I139 (C, O, H^{β})<br>F141 ($H^{\delta 1}$, $H^{\varepsilon 1}$)<br>D147 (H^{α}, O)<br>Y150 ($H^{\beta 1}$, $H^{\beta 2}$, $H^{\delta 2}$, C^{γ})
C	N153 ($H^{\beta 1}$, $H^{\beta 2}$)<br>R156 ($H^{\beta 1}$, $H^{\beta 2}$, $H^{\delta 1}$)<br>Y157 ($H^{\varepsilon 1}$, C^{ζ}, O^{η})<br>F198 ($C^{\delta 2}$, $H^{\delta 2}$, $C^{\varepsilon 2}$, $H^{\varepsilon 2}$)<br>D202 ($H^{\beta 1}$)
D	L125 (N, H^{N}, $H^{\beta 1}$, $H^{\beta 2}$, $H^{\delta 22}$)<br>Y128 ($H^{\beta 2}$)<br>Y162 ($C^{\varepsilon 2}$, $H^{\varepsilon 2}$, H^{η})

[a]Atoms in van-der-Waals contact with xenon were identified in the structure shown in Fig. 5 assuming a van-der-Waals radius of 0.22 nm for xenon.

Table S2 Resonances of PrP showing significant cross peak volume changes after spin labelling[a]

Position of spin label	Residues affected
93	W31, ——, G46, Q52, **G58**, **W65**, **G74**, **G78**, **G82**, —————— , **T95**, **H96**,
107	——, Y38, ——, Q52, G58, ——, G74, —————, G90, Q91, G92, T95, H96,
93	**S97**, **Q98**, **W99**, **N100**, K101, **S103**, K104, **K106**, ————————————
107	S97, Q98, W99, N100, ——————, K104, **K106**, H111, M112, A113, **G114**,
93	————————————————, V122, G123, **L125**, G126, **G127, Y128,**
107	A115, A116, A117, **A118**, **G119**, A120, V122, G123, L125, G126, ———, **Y128**,
93	M129, **L130**, S132, **S135**, ——, H140, **M166**, **Q172**, N173, N174, D178, I182,
107	———, L130, S132, ——, **I138**, ———, **M166**, **Q172**, ——————————
93	**K185**, F198, Y218, S222, **Q223**, Y225, **Y226**, **Q227**, **R228**, **G229**
107	————————, S222, ———, Y225, Y226, Q227, ——— , G229

[a]Only residues with $-\Delta V/V_0 \geq \sigma_0$ are represented. Bold letters, residues with $-\Delta V/V_0 > 2\,\sigma_0$.

Figures

Figure S1 Transient interactions of the unfolded N-terminus with the folded core.

The 20th NMR structure of *hu*PrP of 2KUN is used to show the residues of the N-terminus that possibly interact with the core domain as predicted in this study and by high pressure NMR spectroscopy (Kachel et al., 2004). K101, S103, K104, K106, N108, M109, and M112 show significant pressure effects.

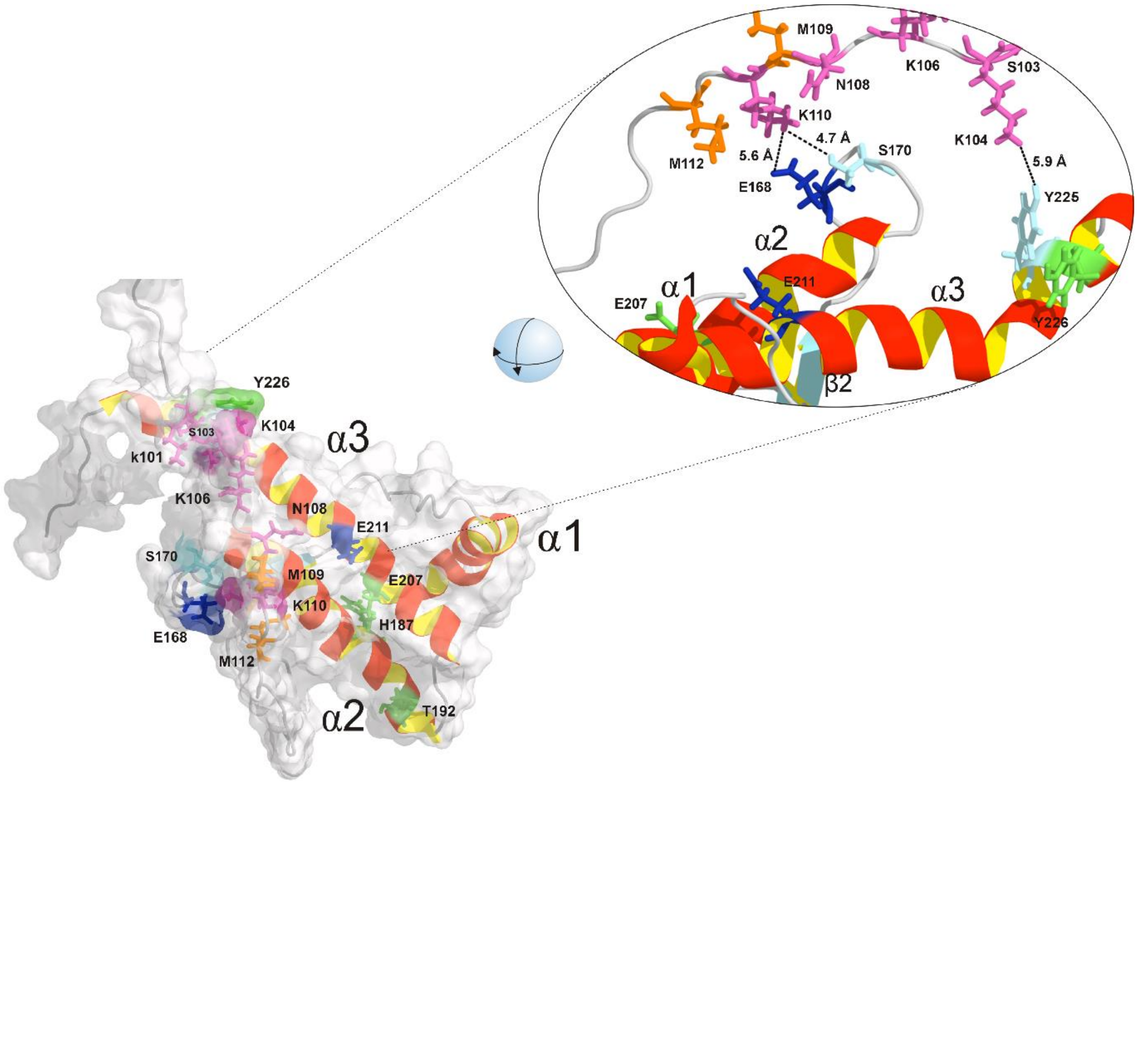

Supplement: Supplementary Information [file srep28419-s1.pdf]
